# Insights into bone morphogenetic proteins in cardiovascular diseases

**DOI:** 10.3389/fphar.2023.1125642

**Published:** 2023-02-23

**Authors:** Di Ye, Yinghui Liu, Heng Pan, Yongqi Feng, Xiyi Lu, Liren Gan, Jun Wan, Jing Ye

**Affiliations:** ^1^ Department of Cardiology, Renmin Hospital of Wuhan University, Wuhan, China; ^2^ Cardiovascular Research Institute, Wuhan University, Wuhan, China; ^3^ Hubei Key Laboratory of Cardiology, Wuhan, China; ^4^ Department of Gastroenterology, Renmin Hospital of Wuhan University, Wuhan, China

**Keywords:** BMP, atherosclerosis, vascular calcification, hypertension, cardiac remodeling, diabetic cardiomyopathy, pulmonary arterial hypertension, hereditary hemorrhagic telangiectasia

## Abstract

Bone morphogenetic proteins (BMPs) are secretory proteins belonging to the transforming growth factor-β (TGF-β) superfamily. These proteins play important roles in embryogenesis, bone morphogenesis, blood vessel remodeling and the development of various organs. In recent years, as research has progressed, BMPs have been found to be closely related to cardiovascular diseases, especially atherosclerosis, vascular calcification, cardiac remodeling, pulmonary arterial hypertension (PAH) and hereditary hemorrhagic telangiectasia (HHT). In this review, we summarized the potential roles and related mechanisms of the BMP family in the cardiovascular system and focused on atherosclerosis and PAH.

## 1 Introduction

Cardiovascular disease (CVD) remains the leading cause of mortality and morbidity worldwide. The mortality of CVD is particularly high in the United States, China, Eastern Europe and India (Roth et al., 2020). Serious CVD results in major economic losses and physical and mental burdens on patients and their families. The survival rates have improved significantly through lifestyle interventions and the widespread use of new technologies and drugs (Fegers-Wustrow et al., 2022). However, the poor prognosis of CVD has not been improved, and the mortality, morbidity, disability and recurrence rates are still very high (Bethel et al., 2018; Arnett et al., 2019).

Bone morphogenetic proteins (BMPs), first discovered in 1965, are a group of evolutionarily conserved secretory proteins that play important roles in growth and development (Bone, 1965). All BMPs belong to the transforming growth factor-β (TGF-β) superfamily except BMP-1 (Kawabata et al., 1998). As research has progressed, in addition to inducing bone and cartilage formation, BMPs have been shown to regulate the development of the embryo, lung, kidney, gastrointestinal system, teeth and other organs (Zhang and Que, 2020). Ablation or overexpression of BMPs usually leads to significant defects or severe pathology. Examples include congenital renal and urinary tract abnormalities (Dudley et al., 1995; Luo et al., 1995; Miyazaki et al., 2000), anophthalmia and microphthalmia (Dudley et al., 1995; Luo et al., 1995), achondrogenesis (Kugimiya et al., 2005), osteoarthritis (Shao et al., 2021), Barrett’s esophagus (Palles et al., 2015), and anemia (Steinbicker et al., 2011). Numerous studies have demonstrated that BMPs are strongly associated with CVD, and research progress has been made recently. BMP activation often results in vascular inflammation, such as atherosclerosis and calcification, whereas BMP suppression is associated with pulmonary arterial hypertension (PAH) and hereditary hemorrhagic telangiectasia (HHT). Of course, this is not absolute. Regulation of BMP signaling may provide a novel strategy for the treatment of CVD.

## 2 Members of the BMP family

To date, the BMP family has been found to have more than a dozen members in vertebrates. BMPs are widely found in pigs, cattle, sheep, rabbits, mice and human embryos, blood cells, kidneys, spleen and other tissues, with high homology between different species. BMPs can be further classified into several subgroups according to their structural homology: the BMP-2/-4 group, the BMP-5/-6/-7/-8 group, the BMP-9/-10 group, and the BMP-12/-13/-14 group (Katagiri and Watabe, 2016). Although BMP-1 can induce bone and chondrogenesis, it is a metalloproteinase that acts as a procollagen C-protease during collagen maturation and does not belong to the TGF-β superfamily (Vadon-Le et al., 2015). At least 6 BMP subtypes (BMP-2/4/5/6/7/10) are expressed in cardiac tissue, and they are also the most studied BMPs in CVD (van Wijk et al., 2007). The BMP family members and their main source and receptors are listed in Table 1.

**TABLE 1:** BMP family members and their main sources and receptors.

Ligands	Alternate names	Source	Type I receptors	Type II receptors	R-Smad	Co-smad
BMP-2	–	ECs, SMCs, Cardiomyocytes	ALK-2/-3/-6	BMPR II	Smad1/5/8	Smad4
				ActR II/IIB		
BMP-3a, 3b	GDF-10	Fibroblasts	ALK-4/-5	ActR II/IIB	Smad2/3	Smad4
BMP-4	–	SMCs, Monocytes, Adipocytes Cardiomyocytes	ALK-2/-3/-5/-6	BMPR II	Smad1/2/5/8	Smad4
				ActR II/IIB		
BMP-5	–	Fibroblasts	ALK-3	BMPR II	Smad1/5/8	Smad4
				ActR II/IIB		
BMP-6	Vgr-1	ECs, SMCs	ALK-2/-3/-6	BMPR II	Smad1/5/8	Smad4
				ActR II/IIB		
BMP-7	OP-1	SMCs	ALK-2/-3/-6	BMPR II	Smad1/5/8	Smad4
				ActR II/IIB		
BMP-8a, 8b	OP-2, OP-3	Fibroblasts	ALK-2/-3/-4/-6	BMPR II	Smad1/5/8	Smad4
				ActR II/IIB	Smad2/3	
BMP-9	GDF-2	ECs, SMCs	ALK-1/-2	BMPR II	Smad1/5/8	Smad4
				ActR II/IIB	Smad2/3	
BMP-10	–	ECs, Cardiomyocytes	ALK-1/-2/-3/-6	BMPR II	Smad1/5/8	Smad4
				ActR II/IIB	Smad2/3	
BMP-11	GDF-11	–	ALK-4/-5/-7	ActR II/IIB	Smad2/3	Smad4
BMP-12	GDF-7	Fibroblasts	ALK-3/-6	BMPR II	Smad1/5/9	Smad4
				ActR II		
BMP-13	GDF-6	Fibroblasts	ALK-3/-6	BMPR II	Smad1/5/8	Smad4
				ActR II/IIB		
BMP-14	GDF-5	–	ALK-6	BMPR II	Smad1/5/8	Smad4
				ActR II		
BMP-15	GDF-9b	–	ALK-4/-5/-6	BMPR II	Smad1/5/8	Smad4
BMP-16	Nodal	–	ALK-4/-7	ActR IIB	Smad2/3	Smad4

GDF: growth and differentiation factor; Vgr-1: Vg1-related protein; OP: osteogenic protein; ECs: endothelial cells; SMCs: smooth muscle cells.

## 3 The BMP signaling pathway

### 3.1 Structural diversity of BMPs

The TGF-β ligands include TGF-β1, TGF-β2, and TGF-β3. They mainly bind to the TGF-β type II receptor (TGFBR II) and then to TGF-β type I receptor (ALK-5) to form a complex. In endothelial cells, TGF-βs can exert distinct effects through another TGF-β type I receptor, ALK-1 (Goumans et al., 2002). In brief, Smad2/3 are generally activated by ALK5, and then they bind to Smad4, enter the nucleus and exert biological effects including inhibiting endothelial cell proliferation and migration. Whereas ALK-1 activation phosphorylates Smad1/5/8, leading to an increase in endothelial cell proliferation and migration, even directly antagonizes ALK-5/Smad signaling (Goumans et al., 2002; Goumans et al., 2003). ALK-1 is also known to cross-talk with the ALK-5, endothelial cells lacking ALK-5 are deficient in TGF-β/ALK1-induced responses (Goumans et al., 2003).

Mature BMP ligands are dimers synthesized by macromolecular pro-proteins that contain an amino-terminal signal peptide, a long pro-peptide, and a carboxy-terminal mature peptide that contains a cystine knot (Goumans et al., 2018). To form active dimeric ligands, these BMP pro-proteins are proteolytically cleaved by proconvertases and then participate in dimerization with another BMP monomer through a covalent disulfide bond (Katagiri and Watabe, 2016). Dimerization mainly occurs intracellularly, and most active BMPs are homodimers (Yadin et al., 2016; Kaito et al., 2018). Heterodimers, such as BMP-2/-5, BMP-2/-6 and BMP-2/-7, sometimes show greater activity than homodimers (Yuan et al., 2011; Guo and Wu, 2012).

Similar to other members of the TGF-β family of ligands, BMP ligand dimers function by promoting the assembly of two serine-threonine kinase receptors on the cell surface (Katagiri and Watabe, 2016). Type I receptors contain seven members, named activin receptor-like kinases (ALK) 1-7, and ALK-1/-2/-3/-6 serve as BMP Type I receptors (BMPR I) (Miyazono et al., 2010). Type II receptors contain three members: the BMP type II receptor (BMPR II), the activin type II receptor (ActR II) and the activin type IIB receptor (ActR IIB) (Rosenzweig et al., 1995). Based on structural homology, BMPR I is divided into two groups: the ALK-3/ALK-6 group and the ALK-1/ALK-2 group (Morrell et al., 2016). Among them, ALK-2 and ALK-3 are widely expressed by diverse cell types, whereas ALK-6 is expressed by fewer cell types and ALK-1 is mainly restricted to endothelial cells (Garcia et al., 2016). BMPR II and ACTR II are ubiquitously expressed *in vivo*, but only BMPR II is highly expressed in endothelial and endocardial tissues (Morrell et al., 2016).

Nevertheless, unlike other TGF-βs, BMPs can bind to type I receptors in the absence of type II receptors (Katagiri and Watabe, 2016). Further research found that the specificity of BMP binding to type I receptors is affected by the presence of type II receptors (Yu et al., 2005). BMP-2 and BMP-4 have high affinity for BMPR II in a complex with ALK-3 or ALK-6 (Goumans et al., 2018). BMP-6 and BMP-7 bind strongly to ActR II with ALK-2 and weakly to ALK-6 (Ning et al., 2019). In endothelial cells, BMP-9 and BMP-10 bind to BMPR II in a complex with ALK-1 or ALK-2 (David et al., 2007; Scharpfenecker et al., 2007). In addition, BMP-14 binds to ALK-6 but not to other type I receptors (Zhang and Que, 2020). BMP-15 binds to ALK-6 and stimulates Smad1/5/8 (Moore et al., 2003). In contrast to other BMPs, BMP-3 and BMP-16 bind to ALK-4 and ALK-5, leading to activation of Smad2 and Smad3 (Katagiri and Watabe, 2016). BMP-3 was reported to bind to ACTR IIB and suppress the activation of BMP-2 and BMP-4 (Kokabu et al., 2012). BMP-11 binds to three BMPR I (ALK-4/-5/-7) and two type II receptors (ActR II/IIB) (Jamaiyar et al., 2017).

### 3.2 BMP signaling pathway

BMPs bind to receptors and activate two major signaling pathways, the canonical signaling pathway and the non-canonical signaling pathway. Canonical BMP signaling has been summarized in previous reviews (Zhang and Que, 2020), (Katagiri and Watabe, 2016), (Morrell et al., 2016). In brief, BMPs bind to specific type I and type II receptors to form heterotetrameric complexes. Next, the type II receptor phosphorylates the type I receptor, which results in the phosphorylation of Smad1/5/8 at the C-terminus (Zhang and Que, 2020). Then, p-Smad1/5/8 binds to Smad4, and the complex enters the nucleus, where it further binds to coactivators or cosuppressors to regulate gene expression (Shi and Massague, 2003).

In addition to canonical BMP signaling, non-canonical signaling pathways play important roles in many biological processes. Various non-canonical pathways, including mitogen-activated protein kinases (MAPKs), c-Jun amino-terminal kinase (JNK), extracellular signal-regulated kinase (ERK), p38 and phosphoinositol-3 kinase (PI3K) and small GTPases, can also lead to the regulation of gene expression (Derynck and Zhang, 2003; NarasimhuluAluganti and Singla, 2020).

BMP signaling is also modulated by coreceptors in the extracellular space (such as noggin, chordin, gremlin, cerberus, and follistatin), intracellular space (such as the E3 ubiquitin ligases Smurf1 and Smurf2 and Smad6/Smad7), and plasma membrane (such as BMP and the activin membrane-bound inhibitors BAMBI and endoglin) (Brazil et al., 2015).

### 3.3 Cross-talk with other signaling pathway

Here we mainly discuss cross-talk between BMP signaling and other signaling pathways related to cardiovascular development and diseases. Previous studies reported that BMP signaling pathways and other signaling pathways exist different levels of cross-talk in vascular development and homeostasis, including vascular endothelial growth factor (Pulkkinen et al., 2021) (VEGF), fibroblast growth factor (Schliermann and Nickel, 2018) (FGF), and Notch (Dahlqvist et al., 2003) and Wnt (Gaussin et al., 2002) signaling.

The use of small interfering RNA revealed that TGF-β1 stimulated VEGF expression by activating ALK-5, TGFBR II, and SMAD2, whereas BMP-9 suppressed it by activating ALK-1, BMPR II, and SMAD1 (Shao et al., 2009). BMP-4 and BMP-7 were reported to repress VEGFA expression to stimulate outflow tract cushion formation (Bai et al., 2013). Both BMPs and FGFs are highly preserved between different species, involved in essential cellular functions, and their ligands vastly outnumber their receptors. On the mesodermal side, FGF-BMP interaction can be observed in cardiogenesis (Schliermann and Nickel, 2018). In the development of heart, the combination of Nodal (BMP-16) and FGF-8 signaling is essential for mesoderm specification and differentiation in the presence of BMP inhibitors, such as chordin, noggin, and follistatin. When the visceral mesoderm is designated, BMP-2 and BMP-4 expressed in the adjacent endoderm act synergistically with FGF-8 and FGF-4 signals to induce heart-specific markers (Nakajima et al., 2009; Meganathan et al., 2015). In human embryonic stem cells, BMPs act together with FGF2, which signals through the ERK/MAPK pathway to drive mesendoderm differentiation (Yu et al., 2011). Many developmental processes are controlled by both the Notch signaling pathway and TGF-β ligands including BMPs. BMP signaling *via* SMAD1/5 activation regulates the expression of Jagged 1 in endothelial cells to transactivate Notch signaling in neighboring cells (Morikawa et al., 2011). BMPs could regulate the Notch transcriptional targets HES1 and HEY1 in mesenchymal lineages to modulate cellular plasticity in a manner independent of canonical Notch activation (Dahlqvist et al., 2003), (de Jong et al., 2004; Itoh et al., 2004). In embryonic endothelial cells, ALK-1activation induced by BMP-9 or BMP-10 and mediated by Smad1/5/8, cooperates with Notch signaling to inhibit angiogenesis (Larrivee et al., 2012). In *Xenopus*, both BMP and Wnt signaling are critical for the activation of genes encoding dorsal fate specification in mesoderm and endoderm (Cui et al., 1996; Zorn et al., 1999). In mouse embryos, Wnt signaling modulates the expression of the BMP target gene Msx2 by inducing the expression of BMP ligands, thereby influencing cell fates in the ectoderm and the neural crest (Hussein et al., 2003). BMP could induce mesendoderm differentiation together with FGF-2 in embryonic stem cells, while requires TGF-β or Wnt signaling (Yu et al., 2011).

Both TGF-β and BMPs can directly activate the ERK, JNK, and p38-MAPK pathways independent of Smad proteins. It was reported that TGF-β could reduce BMP-4 signaling in SMCs, which suggesting a cross-talk between the two signaling pathways (Upton et al., 2013). ATF-2 activation by p38-MAPK was shown to bind to smad1/4 and participate in TGF-β-regulated terminal cardiomyocyte differentiation (Monzen et al., 2001). MAPKs (especially ERK1/2) also phosphorylate the linker of Smad1/5, which almost always blocks Smad1/5 nuclear translocation. As a result, BMP function can be suppressed by several signals that activate RTK/MAPK, including epithelial growth factor (EGF), FGF and insulin-like growth factor (IGF) (Guo and Wang, 2009).

There are also interactions between BMP and TGF-β signaling. Smad4 is shared by both activin/TGF-β and BMP Smad pathways. When the levels of Smad4 are limited, BMP and TGF regulate the activity of each other by competitively binding to smad4 (Candia et al., 1997). It was reported that TGF-β could reduce BMP-4 signaling in SMCs, which suggesting a cross-talk between the two signaling pathways (Upton et al., 2013).

Thus, it seems that the outcome of signaling cross talk is determined by the context of the signaling environment and that multiple signal inputs.

## 4 The BMP family and CVD

### 4.1 The BMP Family and Cardiovascular Development and Differentiation

Cardiac embryonic development is divided into four stages: embryonic stem cell differentiation into lateral plate mesoderm, lateral plate mesoderm differentiation into cardiac progenitor cells, cardiac progenitor cell proliferation and cardiac terminal differentiation.

BMP signaling partly controls the spatiotemporal sequences of pluripotent stem cells and embryonic stem cells differentiation into cardiac lineages, as demonstrated by studies *in vivo* and *in vitro* (Morrell et al., 2016). It was reported that BMP-4 cooperates with FGF2 could induce cardiac mesoderm formation and inhibit endoderm differentiation by activating ERK signaling in a smad1-dependent manner (Faial et al., 2015). On the 3rd to 5th day of human pluripotent stem cell differentiation, the proportion of cardiomyocytes induced by adding BMP4 is significantly increased (Protze et al., 2017). BMP-4 also plays a pivotal role in the differentiation of cardiomyocyte progenitors into cardiomyocytes, but its effect at commitment stages is dependent on a precise balance, with activin A, Nodal, and Wnt signals (Morrell et al., 2016). In addition, cardiac progenitor cells differentiate into epicardial lineages instead of cardiomyocytes if BMP signaling persists for 3 days beyond when cardiac mesoderm is formed by the canonical WNT pathway (Witty et al., 2014).

Both BMP-2 and BMPR-1A are expressed in the cardiac crescent, and BMPR-1A deletion in the cardiac mesoderm leads to loss of cardiac crescent and cardiomyocytes in embryos (Gaussin et al., 2002; Klaus et al., 2007). BMP-2/-4/-5/-6/-7, ALK-2 and BMPR-1A are expressed in the atrioventricular canal (Kruithof et al., 2012). It was reported that only BMP-2 is required for endocardial-to-mesenchymal transition (EMT) and cushion formation in the atrioventricular canal in mouse and chicken models. Furthermore, endocardial-specific ALK-2 deficiency results in decreased smad1/5/8 and smad2/3 phosphorylation, whereas BMPR-1A deletion results in decreased smad1/5/8 phosphorylation (Ma et al., 2005; Wang et al., 2005). Only myocardial-specific deletion of BMPR-1A resulted in cardiac defects, namely reduced atrioventricular cushion size and myocardial thinning (Wang et al., 2005).

BMP-10 is briefly expressed in the ventricular trabecula during midgestation (Chen et al., 2004). In adults, BMP-10 is only expressed in the right atrium, and BMP-10 is induced by myocardin *via* binding to the promoter of BMP-10 (Huang et al., 2012).

### 4.2 The BMP family and atherosclerosis and coronary artery disease (CAD)

Atherosclerosis is characterized by deposition of blood lipids into the intima of the arteries to form atherosclerotic plaques, finally resulting in thickening and hardening of the artery walls and narrowing of the lumen. In addition, atherosclerosis is the main cause of CAD, cerebral infarction and peripheral vascular disease. The pathological mechanism of atherosclerosis is complex, and includes inflammation, lipid metabolic disorders, plaque formation and calcification, macrophage polarization and iron overexpression (Wunderer et al., 2020). Inflammation in the intima results in the migration and proliferation of vascular smooth muscle cells (VSMCs) into the intima, and then, remodeling of the plaque occurs, which further leads to progressive narrowing of vessels. As atherosclerotic plaques develop, the proliferation of smooth muscle cells (SMCs) and the accumulation of some typical types of lipids proceed, and plaque ruptures can occur in advanced stages (Kim et al., 2019). CAD is the further development of atherosclerosis, and its main pathological process is the activation of inflammation and activation of the coagulation system.

TGF-β ligands have been reported to participate in the development of atherosclerosis. It has been reported that the expression of TGF-β ligands, receptors and phosphorylated SMAD2/3 are increased in human atherosclerotic lesions (Bobik et al., 1999; Frostegard et al., 1999; Kalinina et al., 2004). In addition, TGF-β expression was also increased in the plaques of coronary arteries in patients with CAD compared to healthy patients (Wang et al., 1997; Frutkin et al., 2009). This may be due to TGF-β signaling promoting the synthesis of extracellular matrix (ECM) in VSMCs to promote the growth of atherosclerotic lesions (Li et al., 2008). However, several studies have shown that TGF-β protects against atherosclerosis by promoting a stable lesion phenotype by stimulating SMC differentiation and preventing the switch from contractile to proliferative SMCs (Gomez and Owens, 2012). In addition, the expression of TGF-β1 is higher in stable lesions than in unstable lesions (Panutsopulos et al., 2005). Moreover, it was reported that overexpression of TGF-β1 in cardiomyocytes increases the levels of TGF-β1 in the plasma, limits plaque growth and induces plaque stabilization (Frutkin et al., 2009). Furthermore, intraperitoneal administration of anti-TGF-β1 antibody promotes atherogenic changes in the vessel wall in Apoe−/− mice (Tedgui and Mallat, 2001). It was reported that macrophage-specific TGF-β1 overexpression reduces the development of atherosclerotic lesions in Apoe−/− mice and stabilizes existing plaques (Reifenberg et al., 2012). Interestingly, it was reported that TGF-β could reduce BMP-4 signaling in SMCs, which suggesting a cross-talk between the two signaling pathways (Upton et al., 2013).

Previous studies found that the level of BMP-4 was elevated in the aortic wall in an atherosclerosis mouse model (Yao et al., 2009). Macrophage foam cells are recognized as hallmarks of early atherosclerosis (Cheng et al., 2013). Further studies found that BMP-4 could accelerate foam cell formation by BMPR II/Smad signaling (Feng et al., 2014). Neointimal hyperplasia is the major cause of restenosis after percutaneous intervention. One recent study reported that platelet-specific BMP-4 deficiency slows endothelial regeneration and prevents neointimal hyperplasia after carotid wire injury by inhibiting platelet activation, reducing the expression of adhesion molecules and inflammatory responses in mice, and inhibiting endothelial cell proliferation and migration *in vitro* (Jank et al., 2021). The loss of platelet BMP-4 also resulted in decreased platelet-leukocyte aggregated formations in LDLr^−/−^ mice after carotid artery wire injury, and the effect was most pronounced in the early phase after injury, especially in the first 24 h. However, Chen et al. reported that BMP-4 treatment significantly inhibited the migration and recovery of endothelial cells by stimulating the production of ROS, whereas BMP-4 inhibition promoted endothelial regeneration and recovery in an intimal hyperplasia model in SD rats (Li et al., 2022). Another study found that BMP-4 drives inflammation upon low wall shear stress in the arteries during the early stage of atherosclerosis (Souilhol et al., 2020). These studies are consistent with previous views that BMP-4 can induce endothelial inflammation and endothelial dysfunction *in vivo* and *in vitro* (Kim et al., 2013). For the advanced atherosclerosis mouse model, which is induced by an atherogenic diet for approximately 20 weeks, there are also several studies. One study found that BMP-4 expression was decreased in perivascular adipose tissue from mice and humans with atherosclerosis, and scholars further found that both BMP-4 knockdown and adipocyte-specific BMP-4 knockdown aggravated atherosclerotic plaque formation by promoting inflammation and impairing lipid metabolism in brown adipocytes in Apoe−/− mice, while BMP-4 overexpression promoted browning of perivascular adipose tissue and significantly decreased plaques in advanced atherosclerosis (Mu et al., 2021). Interestingly, in a recent clinical study, after adjustment for other cardiovascular risk factors, high circulating BMP-4 levels were negatively correlated with the incidence of multivessel disease in male patients with CAD (Park et al., 2015). However, the correlation was not observed in female patients. Thus, serum BMP-4 can help assess the severity of CAD in male patients. These studies suggest that BMP-4 plays an atherogenic and proinflammatory role in early atherosclerosis. However, the role of BMP-4 in the progression to advanced atherosclerosis depends on other mechanisms, such as different contexts and different tissue and cell types. Further studies are needed to identify the role of BMP-4 in advanced atherosclerosis.

BMP-2 plays a proinflammatory role in early atherosclerosis. It was reported that BMP-2 produced by VSMCs from atherosclerotic lesions promotes monocyte recruitment and inflammation by activating BMPR II in an early atherosclerosis mouse model (Sato et al., 2014). In addition, Johannes et al. reported that BMP-2 could induce human monocyte migration and adhesion to endothelial cells, and prevent monocytes from differentiating into M2 macrophages (Pardali et al., 2018). BMP-2 promoted VSMC migration by activating ERK signaling *in vitro* in a dose-dependent manner (Zhang et al., 2014a). The activation of CD137 signaling was found to increase the expression of BMP-2 and Runx2 in atherosclerotic plaques in Apoe−/− mice (Chen et al., 2017). Furthermore, BMP-2 could upregulate MMP-2 expression in VSMCs and promote the migration and proliferation of VSMCs under hypoxic stimulation (Yang et al., 2018). BMP-2 levels were also increased in the epicardial adipose tissue of CAD patients and were positively associated with the incidence of calcified atherosclerotic plaques (Luna-Luna et al., 2020). Moreover, BMP-2 levels were independently correlated with in-stent restenosis in patients with CAD (Zheng et al., 2017).

The role of BMP-7 in atherosclerosis is controversial. Yu et al. found that serum BMP-7 significantly decreased in patients with CAD but increased in patients with CAD recovery (Yu et al., 2019). One study reported that exogenous BMP-7 significantly decreases plaque formation following the induction of atherosclerosis by inhibiting M1 macrophage differentiation and promoting M2 polarization, whereas macrophage depletion abolishes this beneficial effect in Apoe−/− mice (Singla et al., 2016; Shoulders et al., 2019). On the other hand, Sovershaev et al. reported that BMP-7 increases the incidence of thrombus formation in lipid-rich plaques (Sovershaev et al., 2010). They further reported that BMP-7 promotes thrombus formation by enhancing the adhesion and migration of human monocytic cells, and this effect could be abrogated by inhibitors of BMP signaling (Sovershaev et al., 2016). These studies suggested that the effect of BMP-7 is tightly associated with various immune cells and inflammatory responses.

BMP-11 also exhibited a beneficial role in atherosclerosis. One study found that BMP-11 overexpression reduced the atherosclerotic plaque area in Apoe−/− mice by selectively decreasing the number of macrophages and T lymphocytes within plaques (Mei et al., 2016). In addition, the PPARα-BMP-11 axis was reported to inhibit atherosclerotic plaque formation by protecting vascular endothelial cells from aging and apoptosis in Apoe−/− mice (Dou et al., 2021).

Recently, exogenous BMP-14 treatment was shown to significantly promote the proliferation of epidermal stem cells in a deep partial thickness burn mouse model (Zhao et al., 2021). Zaidi et al. found that BMP-14 expression increased after myocardial infarction, and BMP-14 knockout mice showed increased myocardial apoptosis, worse cardiac function and more fibrosis after myocardial infarction than wild-type mice. *In vitro*, these researchers also found that recombinant BMP-14 improved cardiac function and reduced cardiomyocyte apoptosis by upregulating Smad4 (Zaidi et al., 2010).

Little research has been conducted regarding other BMPs. One single-cell analysis found that BMP-3B (GDF-10) was the key promoter of VSMC phenotypic modulation in the atherosclerotic aortas of Apoe−/− mice on a high cholesterol diet (Brandt et al., 2022). This study demonstrated that BMP-3B might play a detrimental role in atherosclerotic plaque stability. BMP-9 and BMP-10 were reported to promote the recruitment of monocytes to the vascular endothelium in the presence of TNF-α (Mitrofan et al., 2017). However, more experiments with genetically modified mice are needed to confirm the roles of these BMPs.

### 4.3 The BMP family and vascular calcification

Vascular calcification is a pathological process in which abnormal calcium deposits on the walls of blood vessels. Endothelial cells play critical roles in the initiation and development of vascular calcification. During the process, endothelial cells differentiate into chondrocytes and osteoblast-like cells, followed by mineralization of the surrounding matrix (Abdelbaky et al., 2013; Lanzer et al., 2014). Vascular calcification is commonly observed in atherosclerosis, hypertension, diabetic vasculopathy, chronic kidney disease and aging. Previous studies have reported that the BMP family plays critical roles in the vascular system (Li et al., 2008). Numerous studies have reported that most BMPs upregulate the expression of osteogenic genes in several cell types and appear to be both markers and promoters of vascular calcification (Yang et al., 2020).

Numerous studies have demonstrated that BMP-2, BMP-4, and BMP-6 promote the development of vascular calcification (Li et al., 2008; Yung et al., 2015; Zhang et al., 2018a; Wei et al., 2018). In animal models of diabetes, BMP-2 and BMP-4 promote vascular calcification (Bostrom et al., 2011). Erythropoietin was shown to promote VSMC calcification through the activation of the JAK2/STAT3/BMP-2 axis and the NF-KB pathway (He et al., 2019). In addition, trimethylamine N-oxide was found to upregulate the expression of BMP-2 and promote vascular calcification by activating inflammation in chronic kidney disease rats (Zhang et al., 2020). Furthermore, in a microarray analysis of calcified carotid plaques from patients, BMP-2 expression was positively associated with the presence of unstable plaques (Scimeca et al., 2019). Another report showed that aloe-emodin significantly decreased vascular calcification by inhibiting the BMP-2/Smad4/Runx2 pathway both *in vitro* and *in vivo* (Sapkota et al., 2019).

One previous study found that receptor activator of nuclear factor kappaB ligand (RANKL) increased VSMC calcification by promoting BMP-4 expression *in vivo* and *in vitro* (Panizo et al., 2009). Recently, researchers found that calcifications tended to occur in areas with disturbed blood flow in large blood vessels, especially at bifurcated sites, by using computed tomography angiography. These researchers found that the expression of the flow-sensitive transcription factor Krüppel-like factor 2 (KLF2) decreased in the calcified area, and endothelial-specific KLF2 knockdown induced endothelial-to-mesenchymal transition (EndoMT) in endothelial cells and aggravated vascular calcification in Apoe−/− mice, whereas KLF2 overexpression ameliorated vascular calcification by inhibiting disturbed flow-induced activation of BMP-4/Smad1/5 signaling (Huang et al., 2021).

In an early study, BMP-6 overexpression accelerated osteogenic differentiation and mineralization of stem cells (Zachos et al., 2006). In addition, exogenous BMP-6 induced a series of osteoblast-related genes in human mesenchymal stem cells, such as collagen I, osteocalcin and Runx2 (Boskey et al., 2002; Friedman et al., 2006). Recently, it was observed that BMP-6 and ox-LDL synergistically induce osteogenic differentiation and mineralization (Yung et al., 2015). This phenomenon indicates a potential association between BMP signaling, oxidative stress and inflammation in vascular calcification. However, more animal and clinical studies are needed to fully understand the role of BMP-6 in vascular calcification.

BMP-9 induces osteogenic differentiation of VSMCs and promotes hyperphosphate-induced calcification (Zhu et al., 2015). In addition, Fang et al. reported that high phosphate upregulates the expression of BMP-9 in VSMCs (He et al., 2018). These researchers further found that cyclooxygenase 2 (COX-2) treatment enhanced BMP-9-induced calcification in rat VSMCs by activating the Wnt/β-catenin pathway (He et al., 2018).

However, unlike other BMPs, BMP-7 has anti-calcification effects. Vascular calcification is commonly observed in patients with kidney failure and can increase mortality (Rennenberg et al., 2010). BMP-7-deficient mice usually die from perinatal renal failure, whereas recombinant BMP-7 alleviates renal fibrosis and acute renal failure by inhibiting inflammation and apoptosis (Vukicevic et al., 1998; Hruska et al., 2000; Davies et al., 2003). In addition, the expression of BMP-7 was decreased in renal failure (Tobin and Celeste, 2006). In a mouse model of uremia, BMP-7 treatment significantly downregulated osteocalcin and decreased vascular calcification (Davies et al., 2003). Similarly, in a recent study, researchers found that exogenous BMP-7 administration decreased the expression of BMP-2 and Runx2 in aortic tissue and attenuated vascular calcification in chronic uremic rats (Lee et al., 2022a). However, BMP-7 treatment did not protect against vascular calcification that had already occurred in chronic uremic rats (Gravesen et al., 2018). A previous report showed that high levels of vitamin D or phosphate could increase the incidence of vascular calcification, while the function was reversed by recombinant human BMP-7 (Kang et al., 2010). In addition, previous studies have shown that BMP-7 inhibits VSMC proliferation and maintains the VSMC phenotype *in vitro* (Dorai et al., 2000; Dorai and Sampath, 2001).

Nevertheless, no studies concerning other BMPs related to vascular calcification have been reported. Overall, the effects of BMP signaling molecules on vascular calcification are context dependent, tissue dependent, and cell-type specific.

### 4.4 The BMP family and hypertension

Chronic inflammation of the kidney and vascular wall is believed to be the main cause of hypertension. Renal inflammation leads to glomerular injury and impaired urinary sodium excretion, while vascular inflammation leads to endothelial function impairment, increased vascular resistance and wall sclerosis, resulting in chronic hyperactivation of the renin-angiotensin-aldosterone system.

Among the BMP family, BMP-4 is the most studied in relation to hypertension. BMP-4 treatment was shown to significantly elevate blood pressure and lead to endothelial dysfunction by increasing the activity of nicotinamide adenine dinucleotide phosphate (NADPH) oxidase in mice (Miriyala et al., 2006). In addition, another study found that BMP-4 impairs the function of endothelial cells by upregulating oxidative stress-dependent COX-2, and these researchers further found increased BMP-4 and COX-2 expression in the renal arteries of hypertensive rats and humans (Wong et al., 2010). Furthermore, Zhang et al. reported that BMP-4 inhibition improved endothelial function by blocking oxidative stress in db/db mice (Zhang et al., 2014b). This team further found that BMP-4 overexpression upregulated platelet-derived growth factor AA (PDGF-AA) expression, and both PDGF-AA inhibition and neutralization alleviated BMP-4-induced endothelial dysfunction in diabetes mellitus (Hu et al., 2016). Interestingly, hydrogen sulfide was reported to ameliorate endothelial dysfunction in hypertensive rats by inhibiting the BMP-4/COX-2 pathway, which plays a similar role to noggin (Xiao et al., 2016). Recently, endoglin was shown to aggravate endothelial dysfunction and elevate blood pressure by upregulating BMP-4 expression in mice (Gallardo-Vara et al., 2020).

BMP-7 expression was decreased in patients with hypertensive nephrosclerosis (Nguyen et al., 2008; Bramlage et al., 2010). Xia et al. reported that farnesyltransferase inhibition increased the mRNA expression of BMP-7, attenuated myocardial fibrosis and partly improved cardiac remodeling in SHR rats (Li et al., 2013). BMP-7 may play a protective role in hypertension by inhibiting TGF-β signaling and fibrosis, but more animal studies are needed to confirm this hypothesis.

One cross-sectional study showed that circulating BMP-9 levels were negatively correlated with hypertension and CAD (Liu et al., 2019). In a recent study, Wang et al. developed BMP-9 and BMP-10 double knockout mice, and they found dramatic changes, such as a thinner vascular smooth muscle layer, decreased blood pressure and dilated aortic, pulmonary, cardiac arteries and mesenteric arteries (Wang et al., 2021). These researchers further found that BMP-10 overexpression in endothelial cells elevated blood pressure and promoted the formation of contractile VSMCs in mice (Wang et al., 2021). In addition, BMP-10 expression was elevated in the ventricles of hypertensive rats, and BMP-10 mutation was associated with cardiomyocyte hypertrophy and H9C2 proliferation (Nakano et al., 2007; Hirono et al., 2019). The specific role of this molecule needs to be further explored.

### 4.5 The BMP family and myocardial remodeling

Cardiac fibrosis is commonly seen in the cardiac pathological remodeling response to mechanical or biochemical stress, such as hypertension, pressure overload, cardiac inflammation, or myocardial infarction. Cardiac fibrosis is characterized by the induction of the expression of profibrotic growth factors, such as TGF-β, and by the formation of cardiac fibroblasts into myofibroblasts. Activated fibroblasts differentiate into myofibroblasts, which increases their ability to produce ECM proteins. This change leads to increased myocardial stiffness and, ultimately, cardiac dysfunction and heart failure.

The fibrotic process is driven primarily by local myocardial increases in TGF-β (Goumans and Dijke, 2018). It has been reported that TGF-β1 treatment enhances cardiomyocyte apoptosis, increases caspase-3/7 activity and decreases Bcl-2 expression by upregulating Smad-7 (Heger et al., 2011). Activation of TGF-β signaling leads to increased ECM components and collagen production, and results in fibrosis development (Gabriel, 2009). It has been reported that overexpression of TGF-β1 significantly increases the fibrotic area in the left ventricle of mice (Rosenkranz et al., 2002). On the other hand, TGF-β1 depletion or neutralization prevents collagen accumulation after pressure overload and attenuates diastolic dysfunction (Okada et al., 2005; Ellmers et al., 2008).

As previously reported, BMP-4 expression was increased in mice with pathological cardiac hypertrophy and heart failure (HF) patients (Sun et al., 2013). BMP-4 overexpression aggravated cardiomyocyte hypertrophy, apoptosis, and cardiac fibrosis, whereas BMP-4 inhibition alleviated cardiac remodeling in mice (Sun et al., 2013). Recently, Giulia et al. reported that chorordin-like 1 (Chrdl1) plays a protective role in myocardial infarction by inhibiting BMP-4 signaling (Ruozi et al., 2022). Interestingly, Jaeyeaon et al. reprogrammed mouse tail-tip fibroblasts into cells resembling cardiomyocytes, endothelial cells and smooth muscle cells by using BMP-4, microRNA mimic miR-208b-3p and ascorbic acid. These cells formed a tissue-like structure, and implantation of the formed cardiovascular tissue into the infarcted mouse hearts significantly improved cardiac function and promoted cardiac recovery (Cho et al., 2021).

In contrast to BMP-4, several studies have reported BMP-7 as an antifibrotic factor in many tissues, such as the kidneys (Zeisberg et al., 2003; Manson et al., 2011), liver (Kinoshita et al., 2007; Hao et al., 2012; Zou et al., 2019) and lungs (Yang et al., 2013; Liang et al., 2016). Yalei et al. reported that exogenous BMP-7 treatment alleviated myocardial fibrosis and improved cardiac function in rats with myocardial infarction by inhibiting TGF-β1 signaling (Jin et al., 2018). In addition, David et al. reported that BMP-7 treatment inhibits cardiomyocyte hypertrophy *in vitro* and reverses cardiac remodeling under pressure overload (Merino et al., 2016). Consistently, Ana et al. reported that BMP-7-based peptides alleviated pressure overload-induced left ventricle (LV) remodeling (Salido-Medina et al., 2022).

Similar to BMP-7, BMP-9/-10/-11 showed beneficial effects in cardiac remodeling. Previous studies reported that BMP-9 depletion promoted cardiac fibrosis and remodeling in a transverse aortic constriction (TAC) murine model, whereas recombinant BMP-9 treatment reversed the progression of cardiac fibrosis and improved LV function (Morine et al., 2018). In addition, exogenous BMP-10 was reported to promote cardiac repair and improve cardiac function after myocardial infarction in rats (Sun et al., 2014). Consistently, Claire and his colleagues found that double depletion of BMP-9 and BMP-10 resulted in reduced blood pressure and peripheral vascular dilatation, and they further found that BMP-9 depletion alleviated chronic hypoxia-induced pulmonary vascular remodeling and that BMP-10 was associated with hypoxia-induced cardiac remodeling (Bouvard et al., 2022).

BMP-11 overexpression with an adenovirus was reported to alleviate cardiac ischemia‒reperfusion injury by enhancing mitochondrial biogenesis and telomerase activity in rats (Chen et al., 2021). These researchers also found that BMP-11 depletion resulted in the opposite effect in mice (Chen et al., 2021). Furthermore, BMP-11 overexpression reduced cardiomyocyte apoptosis and improved cardiac function, thus enhancing myocardial regeneration after ischemia‒reperfusion injury in aging mice (Du et al., 2017).

### 4.6 The BMP family and HF

HF is the final stage in the development of CVD. Kevin et al. reported that BMP-9 expression significantly increased in the circulation and LV of patients with HF (Morine et al., 2018). These researchers also found that exogenous BMP-9 treatment limits the development of cardiac fibrosis and improves the function of the LV; in contrast, BMP-9 depletion promoted cardiac fibrosis and aggravated cardiac dysfunction in a murine model of HF (Morine et al., 2018). These results suggest that BMP-9 plays an antifibrotic role in the development of HF.

One study reported that BMP-6 plasma levels significantly increased in chronic HF patients and were positively associated with the severity of HF (Banach et al., 2016). BMP-10 activation improved cardiac function after exposure to isoproterenol infusion by enhancing Smad and Stat3 signaling (Qu et al., 2019).

### 4.7 The BMP family and diabetic cardiomyopathy (DMCM)

DMCM is characterized by myocardial structural abnormalities, including cardiac fibrosis, cardiomyocyte hypertrophy, and apoptosis, that ultimately lead to cardiac dysfunction. Previously, high glucose was shown to induce BMP-2 secretion *in vitro* (Chen et al., 2006). In addition, BMP-2 levels were significantly increased in patients with type 2 diabetes (T2DM) (Zhang et al., 2015). Recently, it was reported that BMP-2 expression was decreased in patients with chronic HF with diabetes, and BMP-2 levels were negatively correlated with the levels of ANP and BNP in patients with CHF and diabetes (Zhang et al., 2021). BMP-2 expression exhibited a similar trend in a rat model of myocardial damage and diabetes, and researchers also found that exogenous BMP-2 alleviated doxorubicin- and high glucose-induced inflammation and pyroptosis *in vitro* (Zhang et al., 2021). However, the direct effect of BMP-2 on diabetes *in vivo* has not been reported thus far.

Mitsuhisa et al. reported that BMP-4 expression was upregulated in the aortas of diabetic Apoe−/− mice (Koga et al., 2013). In addition, increased BMP-4 expression resulted in excessive oxidative stress and endothelial dysfunction in the aortas of diabetic mice (Liu et al., 2021). However, data from previous clinical experiments reported that serum BMP-4 levels were significantly decreased in patients with diabetes (Yurekli et al., 2018). The direct effect of BMP-4 on the hearts of diabetic animals has not been reported.

Recently, Ibrahim et al. reported that exogenous BMP-7 alleviates inflammation, attenuates cardiac remodeling and improves LV function in diabetic mice (Urbina and Singla, 2014; Elmadbouh and Singla, 2021). Consistently, Mitchel et al. found that BMP-7 overexpression by a recombinant adeno-associated viral vector decreased cardiac fibrosis, cardiomyocyte hypertrophy and cardiomyocyte apoptosis and improved cardiac function in a murine model of DMCM (Tate et al., 2021).

### 4.8 The BMP family and aortic dissection (AD)

Acute AD is one of the most common thoracic aortic emergencies and may quickly become fatal without early diagnosis and appropriate management. The initiating event of thoracic AD may be related to a medial hematoma bursting inward through the media or the development of an intimomedial hematoma.

It has been hypothesized that TGF-β stimulates the formation of aortic aneurysm (Gomez et al., 2009). At present, there is little literature on BMP involvement in AD. In a recent study, BMP inhibition by LDN-193189, a potent selective BMP type I receptor (BMPR I) inhibitor, significantly reduced maximal ascending aorta diameter and systolic blood pressure in 3-aminopropionitrile fumarate (BAPN)- and Ang II-treated mice (Chen et al., 2022). Most importantly, LDN-193189 treatment decreased the incidence of AD by 70% (Chen et al., 2022). BMP-11 levels were decreased in thoracic aortic tissues in a mouse model of thoracic AD; in contrast, both BMP-11 treatment and BMP-11 overexpression inhibited SMC phenotypic transition and reduced aortic lesions (Ren et al., 2021).

### 4.9 The BMP family and doxorubicin (DOX)-Induced cardiotoxicity

DOX is one of the most widely used antitumor anthracycline antibiotics owing to its potent activity against a variety of neoplastic diseases. However, the clinical application of DOX is limited by various side effects, especially the most severe dose-dependent and cumulative cardiotoxicity. Inflammation, oxidative damage and apoptosis play important roles in DOX-induced cardiac injury.

Previously, BMP-2 was reported to alleviate DOX-induced cardiomyocyte injury *in vitro* (Izumi et al., 2006). Recently, Peng et al. reported that DOX treatment decreased BMP-10 expression in mouse hearts and that cardiac-specific BMP-10 inhibition aggravated oxidative stress and apoptosis and worsened cardiac function, whereas cardiac-specific BMP-10 overexpression and exogenous BMP-10 supplementation ameliorated DOX-induced cardiac dysfunction by activating STAT3 signaling (An et al., 2022).

### 4.10 The BMP family and atrial fibrillation (AF)

Previously, Xin et al. reported that BMP-7 played a protective role in cardiac functions in a murine AF model (Chen et al., 2016). Jasmeet et al. reported that BMP-10 was a potent biomarker in predicting recurrent AF after AF ablation (Reyat et al., 2020). Recently, in a large clinical study of AF, researchers found that plasma BMP-10 levels showed the strongest positive association with the risk of ischemic stroke regardless of whether patients were on anticoagulants, even after adjustment for age, renal function, and all clinical characteristics (Hijazi et al., 2022). However, these studies are not sufficient to identify the specific role of BMPs in AF, and more research is needed.

### 4.11 The BMP family and cardiac aging

One study found that BMP-2 treatment decreases the migration of endothelial cells in both young and old mice, while endothelial cells from old mice showed better migration than those from young mice (Dadwal et al., 2021).

Most studies support that systemic BMP-11 levels decline with age and that the effect of BMP-11 on body weight is similar to that of myostatin (Loffredo et al., 2013; Poggioli et al., 2016). However, several researchers have found that BMP-11 levels do not decline throughout aging (Harper et al., 2016; Schafer et al., 2016; Garbern et al., 2019). In an early study, BMP-11 overexpression was found to reverse aging-related cardiac hypertrophy in mice (Loffredo et al., 2013). However, Smith and others repeated this research and surprisingly found that BMP-11 treatment had no effect on aging-related cardiac hypertrophy (Smith et al., 2015). Interestingly, Poggioli et al. also repeated this study and found that BMP-11 treatment reduced heart weight in both young and aging mice (Poggioli et al., 2016). More recently, researchers found that exogenous BMP-11 improves metabolic homeostasis and promotes recovery after ischemic stroke in aging mice (Hudobenko et al., 2020; Walker et al., 2020). Importantly, in a clinical study, Kristoff et al. reported that circulating BMP-11 levels decreased in older individuals and were negatively associated with the risk of cardiovascular events and mortality (Olson et al., 2015). Collectively, the role of BMP-11 in cardiac aging is controversial, and more clinical and animal studies are needed.

### 4.12 The BMP family and PAH

The BMP family plays an important role in the development and progression of several vascular diseases, including HHT and PAH. PAH is a subtype of pulmonary hypertension typically characterized by elevated pulmonary arterial pressure and pulmonary vascular resistance and can lead to right HF and death (Ruopp and Cockrill, 2022). In recent years, with the unremitting exploration and gradual understanding of PAH by scientists, the prognosis of patients with PAH has substantially improved, and the survival time has also been significantly prolonged. In particular, the emergence of pulmonary artery targeted therapy has strongly improved the survival rate of patients (Boucly et al., 2021). PAH is very dangerous due to its rapid progression, high mortality and morbidity, and poor prognosis (Hassoun, 2021).

The role of BMP-9 in PAH is controversial. Previous studies have shown that gene mutations in the BMP pathway are important causes of hereditary PAH (Hodgson et al., 2020; Guignabert and Humbert, 2021). Dysregulation of BMP signaling causes phenotypic switching of smooth muscle cells, fibroblasts and endothelial cells, which is the pathological mechanism of PAH (Morikawa et al., 2019; Yeo et al., 2020). In particular, mutation of BMPR Ⅱ was observed in 70% of hereditary PAH and 25% of idiopathic PAH patients (Happe et al., 2020; Maron et al., 2021). Guo et al. reported that BMPR II is a low-affinity receptor; thus, mutations affect the expression of BMPR II in the lung vasculature (Guo et al., 2022). Recently, researchers found that BMPR II mutations reduced the expression of IL-15 in human pulmonary arterial endothelial cells, thus resulting in a decrease in NK cells (Hilton et al., 2022). These researchers further found that NK-deficient IL-15 KO mice developed more severe PAH in a rat model (Hilton et al., 2022). Long et al. injected recombinant BMP-9 into mice to enhance the biological effects of BMPR Ⅱ in the vascular endothelium, and they found that BMP-9 treatment successfully reversed PAH by preventing endothelial cell apoptosis and permeability in a transgenic mouse model and in a monocrotaline-induced rat model (Long et al., 2015). In another study, Theilmann et al. found that endothelial BMPR II knockdown switches the effect of BMP-9 from suppressing endothelial cell proliferation to promoting proliferation, and BMP-9-induced proliferation with BMPR II loss is linked to the prolonged induction of the canonical BMP target ID1 (Theilmann et al., 2020). In addition, the interaction between endothelin-1 (ET-1) and BMPR Ⅱ could induce the proliferation of pulmonary arterial smooth muscle cells in PAH (Maruyama et al., 2015; Maruyama et al., 2022). In contrast, Ly et al. reported that both BMP-9 knockdown and BMP-9 neutralization prevent chronic hypoxia-induced pulmonary hypertension (Tu et al., 2019). These researchers further found that BMP-9 knockdown mice had lower levels of ET-1 and higher levels of 2 potent vasodilator factors, apelin and adrenomedullin (ADM), suggesting that BMP-9 is a key regulator in the balance of key vascular tone regulators *in vivo* and *in vitro* (Tu et al., 2019). Conflicting studies suggest that the role of BMP-9 in PAH is complex and involves many ligands and many receptor combinations.

In clinical studies, the levels of BMPR Ⅱ and BMP-4 in the serum of PAH patients were decreased, which may be associated with endothelial cell injury (Maruyama et al., 2015). In another clinical study, circulating levels of BMP-7 were associated with the mortality of patients with PAH (Liu et al., 2016). Furthermore, BMP-7 expression was decreased in a hypoxia-induced PAH rat model, whereas recombinant BMP-7 treatment significantly reduced EndoMT *in vivo* and *in vitro* (Zhang et al., 2018b).

Mutations in the genes encoding endoglin, ALK3 and BMP-9/GDF-2 have also been reported to be associated with PAH (Machado et al., 2015; Hodgson et al., 2020; Yung et al., 2020). Joshua et al. found that PAH patients with GDF-2 mutations had lower levels of BMP-9 and BMP-10 and reduced BMP activity (Hodgson et al., 2020). Recently, researchers found that ALK3 suppressed excessive EndoMT by inducing the interaction between ID2 and ZEB1 (Lee et al., 2022b).

Tacrolimus and berberine treatment alleviated right ventricular fibrosis and restored right ventricular function by enhancing BMP signaling *in vivo* (Chen et al., 2019; Boehm et al., 2021). Sotatercept is a novel fusion protein that binds to activin and growth differentiation factors to restore the balance between the growth-promoting and growth-inhibiting signaling pathways of BMPR II. In a recent clinical study, researchers found that in adults with pulmonary hypertension and background therapy, sotatercept reduces pulmonary vascular resistance (Humbert et al., 2021). In addition, the 6-min walk distance and NT-proBNP level were also improved (Humbert et al., 2021). These studies suggested that enhancing BMP signaling may be a novel direction for the treatment of PAH (Dunmore et al., 2021). However, direct treatment with recombinant BMPs for clinical application is difficult due to its high cost. Small molecule agonists of BMP pathways may be a future clinical approach to solve this problem.

### 4.13 The BMP Family and HHT

HHT is a rare autosomal dominant disorder characterized by cutaneous mucosal telangiectasia and arteriovenous malformation of the gastrointestinal tract (Ruiz-Llorente et al., 2017). HHT is more common in the brain, lung, gastrointestinal tract and liver. At present, there is no effective treatment in the clinic, and symptomatic treatment is the main treatment. With the identification of gene mutations and many animal studies, inhibition of the TGF-β/BMP signaling pathway was found to be the main cause of HHT. The typing of HHT depends on the genotype of the mutation. Most HHT is caused by mutations in endoglin (HHT-1) and ALK1 (HHT-2) (Ruiz-Llorente et al., 2019). Smad 4 mutations are present in juvenile polyposis-HHT syndrome (JP-HHT) (Ola et al., 2018). BMP-9/GDF2 mutations are present in HHT-5 (Upton et al., 2022).

Endoglin is a coreceptor for BMP-9 and BMP-10, which are highly expressed in endothelial cells. Simon et al. found that endothelial-specific endoglin depletion results in a significant reduction in mean aortic blood pressure due to arteriovenous malformations in the peripheral vasculature (Tual-Chalot et al., 2020). These researchers further found that endoglin maintains the balance of VEGF signaling in quiescent endothelial cells, while endoglin depletion results in abnormal endothelial proliferation in peripheral arteriovenous (Tual-Chalot et al., 2020). ALK1 is the receptor for BMP-9 and BMP-10. BMP-9 treatment was reported to inhibit vascular hyperplasia by blocking the ALK1/PI3K pathway (Alsina-Sanchis et al., 2018). This finding suggests that PI3K inhibitors may serve as potent therapeutic agents for HHT2.

BMP-10 mutants result in skin and liver vascular abnormalities due to high output HF (Capasso et al., 2020). In a recent study, researchers deleted ALK1 in different subsets of endothelial cells and found that ALK1 deletion in capillaries and veins resulted in severe arteriovenous malformations by disturbing flow-migration coupling (Park et al., 2021). In another recent study, liver sinusoidal endothelial cell-specific ALK1 deletion resulted in increased hepatic vascular malformations and posthepatic flow in mice (Schmid et al., 2022).

In a recent case report of one HHT family, circulating BMP-9 levels were significantly lower than those in controls (Balachandar et al., 2022). These researchers also found that GDF-2 mutations disrupt correct cleavage of BMP-9, thus resulting in loss of the active mature BMP-9 dimer (Balachandar et al., 2022). In another case report, Sommer et al. found that low-dose tacrolimus treatment reduced the likelihood of bleeding in a patient with HHT (Sommer et al., 2019).

## 5 Discussion

Since BMP was extracted from bone, an increasing number of functions of BMP have been discovered by scholars, and research on BMP in the cardiovascular direction has gradually improved. However, its specific role in the occurrence and development of CVDs has not been conclusively identified, and its initiation and induction factors in the pathogenesis of CVDs have not been fully explored. In this review, we summarized the structure, signaling pathways and roles of BMP family members in CVDs. The regulation of BMP family members in CVD is summarized in Table 2. BMPs play important roles in several CVDs. Several genomic mouse models are listed in Table 3. Although recombinant BMP proteins and neutralizing antibodies have been shown to be effective *in vitro* and *in vivo*, the economic burden on the public is substantial due to the large quantities of ligand that must be used in the clinic. Therefore, the development and acquisition of cheaper small molecules to regulate BMP signaling may be a novel direction in the treatment of CVD. However, most importantly, the safety of drugs regulating BMP expression needs further study.

**TABLE 2:** Regulatory effects of BMP family members on cardiovascular diseases.

Disease	atherosclerosis、CAD	Vascular calcification	Hypertension	Myocardial remodeling	Heart failure	DMCM	Aortic dissection	Doxorubicin-induced cardiotoxicity	Cardiac aging	PAH
BMP-2	Aggravate	Aggravate	–	–	–	–	–	–	–	–
BMP-4	Controversial	Aggravate	Aggravate	Aggravate	–	–	–	–	–	–
BMP-6	–	Aggravate	–	–	–	–	–	–	–	–
BMP-7	Controversial	Alleviate	–	Alleviate	–	Alleviate	–	–	–	Alleviate
BMP-9	–	Aggravate	–	Alleviate	Alleviate	–	–	–	–	Controversial
BMP-10	–	–	Aggravate	Alleviate	–	–	–	Alleviate	–	–
BMP-11	Alleviate	–	–	Alleviate	–	–	Alleviate	–	Controversial	–
BMP-14	Alleviate	–	–	–	–	–	–	–	–	–

CAD: coronary artery disease; DMCM: diabetic cardiomyopathy; PAH: pulmonary arterial hypertension.

**TABLE 3:** The list of genomic mouse models.

Gene/Protein	Mutation	Tissue	Phenotype
BMPR2/BMPR II	Null	Global	Embryonic lethality owing to gastrulation defect Beppu et al. (2000)
	Heterozygous	Global	Mild susceptibility to PAH Beppu et al. (2004)
	N-terminal exon 2 deletion	Global	Outflow tract and septation defects Delot et al. (2003) and susceptibility to hypoxic PAH Frank et al. (2008)
	Dominant negative transgene	Smooth muscle	PAH and pulmonary vascular remodeling West et al. (2004)
ACVRL1/ALK-1	Null	Global	Midgestational lethality owing to cavernous vessel defects, arterial dilatation, smooth muscle recruitment defects Oh et al. (2000); Urness et al. (2000)
	Heterozygous	Global	Arteriovenous malformations with HHT-like features Urness et al. (2000); Srinivasan et al. (2003)
ENG/Endoglin	Null	Global	Embryonic lethality owing to yolk-sac angiogenesis defect Li et al., 1999; Arthur et al., 2000
GDF-2/BMP-9	Null	Global	Viable with abnormal lymphatic development and drainage Levet et al. (2013)
BMP-10/BMP-10	Null	Global	Embryonic lethality with diminished cardiomyocyte proliferation Chen et al. (2004)
BMP-6/BMP-6	Null	Global	Massive iron overload owing to defective hepcidin expression Meynard et al. (2009)
BMP-2/BMP-2	Null	Global	Embryonic lethality with defects in extra-embryonic and cardiac development Zhang and Bradley (1996)
BMP-4/BMP-4	Null	Myocardium	Defects in atrioventricular septation Jiao et al. (2003)
ACVR1/ALK-2	Null	Endocardial cells	Defects in atrioventricular septa and valves Wang et al. (2005)
BMPR1A/ALK-3	Null	Global	Early embryonal lethality Mishina et al., 1995
	Null	Smooth muscle	Cardiac structural and angiogenesis defect El-Bizri et al. (2008a)
	Heterozygous or null	Patchy smooth muscle	Decreased susceptibility to hypoxic PAH and increased proximal pulmonary arterial stiffness El-Bizri et al. (2008b); Vanderpool et al. (2013)
